# Real-world outcomes treating patients with advanced cutaneous squamous cell carcinoma with immune checkpoint inhibitors (CPI)

**DOI:** 10.1038/s41416-020-01044-8

**Published:** 2020-09-01

**Authors:** Glenn J. Hanna, Emily S. Ruiz, Nicole R. LeBoeuf, Manisha Thakuria, Chrysalyne D. Schmults, James A. Decaprio, Ann W. Silk

**Affiliations:** 1 Department of Medical Oncology, Dana-Farber Cancer Institute, Boston, MA USA; 2 Department of Medicine, Brigham and Women’s Hospital and Harvard Medical School, Boston, MA USA; 3 Center for Cutaneous Oncology and Dermatology, Dana-Farber/Brigham & Women’s Cancer Center, Boston, MA USA

**Keywords:** Squamous cell carcinoma, Cancer immunotherapy

## Abstract

**Background:**

Immunotherapy has revolutionised the treatment of advanced cutaneous squamous cell carcinoma (cSCC). It is important to understand both safety and efficacy in a real-world and trial-ineligible cSCC population. We aimed to evaluate safety, efficacy and molecular insights among a broader cSCC population, including immunosuppressed patients, treated with immune checkpoint inhibitors (CPI).

**Methods:**

We present a cohort of advanced cSCC patients (*n* = 61) treated from 2015 to 2020 evaluating the best overall response (BOR) (RECISTv1.1) to CPI therapy, immune-related adverse events (irAEs) and tumour mutational burden (TMB) to correlate with outcomes. A validated geriatric scoring index (CIRS-G) was utilised to assess comorbidities among patients ≥75. These data were compared with published clinical trial results among the broader cSCC population.

**Results:**

BOR to CPI was lower among the entire cohort when compared with trial data (31.5 vs. 48%, *P* < 0.01), with higher rates of progression (59 vs. 16.5%, *P* < 0.01), regardless of immunosuppression history or age. Grade 3+ irAEs were more common among responders (*P* = 0.02), while pre-treatment lymphocyte count and TMB predicted response (*P* = 0.02).

**Conclusions:**

We demonstrate comparatively lower response rates to CPI among real-world cSCC patients not explained by older age or immunosuppression history alone. Immune-related toxicity, absolute lymphocyte count and TMB predicted CPI response.

## Background

As the average lifespan continues to increase, so does the incidence of cutaneous squamous cell carcinoma (cSCC) with approximately one in five older adults diagnosed in their lifetime.^1,2^ While the management for curable disease centres on surgical resection and adjuvant radiotherapy,^3^ more cases of inoperable or incurable, metastatic cSCC are a reality as the global population ages. Elderly patients (herein defined as individuals 75 years and older) and those with a history of immunosuppression are often significantly underrepresented in clinical trials^4,5^ with challenges attributed to age-related organ function decline, pharmacokinetics, comorbidity and toxicity risk often cited.

In the last several years, the management of advanced cSCC has been revolutionised with the introduction of checkpoint inhibitor (CPI) immunotherapy. Agents targeting programmed cell death 1 protein (PD-1) or its ligand (PD-L1) have demonstrated exceptional, often durable response rates (approaching 50%), which translate to improved survival among cSCC patients in the advanced disease setting.^6^ These findings led to U.S. Food and Drug Administration (FDA) approval of cemiplimab (an anti-PD-1 agent) in September 2018. While prior cSCC studies using CPIs included some patients 75 years and older, patients with immune compromise or immunosuppression needs (human immunodeficiency virus [HIV], haematologic malignancies and solid organ transplant [SOT] recipients) and active autoimmune conditions (requiring therapy within the last 5 years) were excluded. It remains unclear if the benefit and toxicity profile of these agents are generalisable to our oldest or most vulnerable patients in the face of incurable skin cancer.

While recent series have emerged that report comparable efficacy and favourable safety when using immunotherapy among challenging and trial-ineligible patients with other solid tumour types,^7–9^ we present real-world data about efficacy, safety and molecular features among cSCC patients treated with CPI, focusing on elderly patients and those with a history of immunosuppression.

## Methods

### Study cohort

The institutional electronic health record was queried by diagnosis code to identify patients treated with a CPI (PD-1 blockade agents pembrolizumab, cemiplimab or nivolumab) in the Centers for Head and Neck and Cutaneous Oncology between August 2015 and January 2020 following expedited institutional review board approval for existing protocols. Patients were included if they carried a diagnosis of locoregionally advanced, incurable or distant metastatic cSCC (distant disease defined patients with disease beyond regional nodal involvement). Demographics, clinicopathologic features including a history of immune compromise or immunosuppression needs (HIV, haematologic malignancies, SOT recipients and autoimmune disorders), absolute lymphocyte count (ALC, reference normal range: 210–2740 cells/µL), treatment history and outcomes were recorded.

### Efficacy, safety and outcomes

The best overall response (BOR) to CPI therapy was confirmed using Response Evaluation Criteria in Solid Tumors (RECIST) version 1.1.^10^ Immune-related toxicity was assessed using Common Terminology Criteria for Adverse Events (CTCAE) v5.0 scoring.^11^ Progression-free survival (PFS) was defined as the time from the start of CPI therapy to the earlier date of progression and date of death (or, if alive and without evidence of progression, censored at the date of the last follow-up). Overall survival (OS) was determined from the date of initiation of CPI therapy to death or censored at the last follow-up (OS from the date of initial diagnosis was only used when specified).

### Assessing comorbidity

Each patient in the cohort who initiated CPI therapy at age 75 or older was evaluated using the validated Cumulative Illness Rating Scale for Geriatrics (CIRS-G),^12,13^ which assigns a score to each of 14 specific organ systems (scale: 0–4) and an overall combined score (scale: 0–56) to assess baseline comorbidity status and general health. Increasing scores in each section and cumulatively provides a sensitive capture of all coexisting diseases or morbidity among an individual geriatric patient.^14^ Scoring was performed using health record review by a clinical oncologist trained in the scale, but blinded to clinical or CPI outcome data, and was based on disease status within 3 months of the date of CPI initiation.

### Tumour molecular profiling

Molecular profiling was performed to understand certain genetic changes within tumours among the cohort. All in-house sequenced patients (55/61, 90%) separately consented to our institutional genomic profiling study,^15^ and a CLIA-certified laboratory performed molecular testing. Sequencing required availability of >20% tumour on haematoxylin and eosin slides. Qiagen kits were used to isolate and Qubit dsDNA to detect (Invitrogen) and quantify isolated DNA, as previously described.^16^ Libraries were prepared and hybridised to a custom RNA bait set (Agilent SureSelect) targeting the full coding regions of 447 genes and 60 selected intronic regions (OncoPanel v3). Hybrid-capture libraries were sequenced on an Illumina HiSeq 2500 using 2 × 100 paired-end reads and analysed following quality control measures. A subset of patients (*n* = 13, 24%) had tumour sequencing using the validated clinical next-generation sequencing FoundationOne CDx® assay^17^ with processing at Foundation Medicine clinical laboratory (Cambridge, MA). Tumour mutational burden (TMB) was evaluated as tumours with high numbers of mutations may be more likely to respond to immunotherapy. TMB was determined by the number of non-synonymous somatic mutations that occurred per megabase of exonic sequence data across all genes on each panel.

### Statistical analysis

A Wilcoxon rank-sum test and Chi-square test statistic were used to compare continuous and categorical variables among age subgroups, respectively. Multivariate Cox proportional hazard modelling was used to evaluate the impact of clinicopathologic features on survival (only performed if there were more than *n* = 10 patients in each subgroup); a test for interaction was performed among all univariate predictors that met the threshold for statistical significance. The Kaplan–Meier method was applied using log-rank testing to evaluate survival outcomes. A Chi-square test statistic was used to compare response rates among our cohort and previously published immunotherapy-treated populations.^6^ A Mann–Whitney *U* test was used to compare median CIRS-G scores between age and immune subgroups, as well as TMB or ALC by response to CPI therapy (a Kruskal–Wallis test compared median TMB among age subgroups). All statistical tests were two-sided, and a *P* value < 0.05 was deemed significant (Stata 14.2, College Station, TX).

## Results

### Study cohort

Among 61 patients, median age of the cohort was 75 (range: 42–95) at the start of CPI therapy, with 20% of the cohort 85 years of age or older when starting a CPI for cSCC (Table 1). The majority of patients were male (80%), former or current smokers (59%) and had distant metastatic disease (77%; 24 distant or non-regional nodes, 11 pulmonary, 6 osseous, 3 subcutaneous, 2 central nervous system and 1 hepatic). Nineteen of 61 (31%) had a history of immunosuppression or a significant autoimmune condition (requiring systemic immunosuppression in the last 5 years) prior to CPI exposure (Supplementary Table 1). All received single-agent PD-1 blockade with 37 (61%) in the first-line (1L) setting for advanced, incurable or metastatic disease and 24 (39%) in the second-line (2L) setting after chemotherapy and/or epidermal growth factor receptor (EGFR) inhibitor therapy use in the 1L. Following CPI discontinuation due to progression or intolerance, 16 (26%) went on to subsequent systemic therapies. There was no difference in clinicopathologic characteristics when separating by age (cut-off 75 or older), with the exception of a lower initial ALC among the elderly subgroup (*P* = 0.03).

**Table 1:** Demographics and clinical characteristics of patients with advanced cutaneous squamous cell carcinoma (cSCC) separated by age of initiation of an immune checkpoint inhibitor (CPI).

Characteristic	All (%)^a^, *N* = 61	Age 75+, *N* = 36	Age < 75, *N* = 25	*P* value^b^
*Age at CPI initiation (median, y)*	75 (42–95)	81 (75–95)	61 (42–69)	0.65
≥70 years of age	36 (59)	36 (100)	0	
≥80 years of age	20 (33)	20 (56)	–	
≥90 years of age	3 (5)	3 (8)	–	
*Sex*				0.71
Male	49 (80)	29 (81)	20 (80)	
Female	12 (20)	7 (19)	5 (20)	
*ECOG performance status*				0.83
0	13 (21)	10 (28)	3 (12)	
1	34 (56)	19 (52)	15 (60)	
2	11 (18)	6 (17)	5 (20)	
3	3 (5)	1 (3)	2 (8)	
*Smoking history*				0.59
Never or <10 pack-years	25 (41)	11 (31)	14 (56)	
Former or current (≥10 pack-years)	36 (59)	25 (69)	11 (44)	
*Immune suppression*				0.32
None	42 (69)	24 (67)	18 (72)	
HIV	2 (3)	1 (3)	1 (4)	
Solid organ transplant history	5 (8)	2 (6)	3 (12)	
Non-Hodgkin lymphoma	7 (11)	4 (11)	3 (12)	
Autoimmune disease	5 (8)	5 (14)	0	
*Primary site of disease*				0.11
Head and neck	17 (28)	3 (8)	14 (56)	
Limb	25 (41)	21 (58)	4 (16)	
Torso	16 (26)	11 (31)	5 (20)	
Unknown	3 (5)	1 (3)	2 (8)	
*Initial staging at diagnosis*^c^				0.37
Stages I and II	17 (28)	10 (28)	7 (28)	
Stages III and IV	44 (72)	26 (72)	18 (72)	
*Pathologic differentiation*				
Well differentiated	9 (15)	5 (14)	4 (16)	
Moderately differentiated	29 (48)	17 (47)	12 (48)	
Poorly differentiated	21 (34)	12 (33)	9 (36)	
Not known	2 (3)	2 (6)	0	
Baseline absolute lymphocyte count^d^	820 (200–88600)	755 (200–6710)	940 (280–88600)	0.03
*Initial treatment regimen*				0.81
Radiation alone	4 (7)	3 (8)	1 (4)	
Surgery alone	12 (20)	8 (22)	4 (16)	
Surgery + radiation	14 (23)	9 (25)	5 (20)	
Surgery + CRT	13 (21)	7 (19)	6 (24)	
Definitive CRT	5 (8)	2 (6)	3 (12)	
Chemotherapy	9 (15)	3 (8)	6 (24)	
Immune checkpoint blockade	4 (7)	4 (11)	0	
*Extent of advanced disease*				0.84
Locoregional only	14 (23)	9 (25)	5 (25)	
Distant metastases^e^	47 (77)	27 (75)	20 (75)	
*CPI line of therapy*				0.43
First	37 (61)	21 (58)	16 (64)	
Second and beyond	24 (39)	15 (42)	9 (36)	

*ECOG* Eastern Cooperative Oncology Group, *CRT* concurrent chemoradiation.

^a^Except for age.

^b^Wilcoxon rank-sum test for continuous variables and Chi-square testing for categorical variables, *P < 0.05, two-sided.

^c^American Joint Committee on Cancer (AJCC) 7th edition staging (2010).

^d^Recorded at the start of CPI initiation, reference normal range: 210–2740 cells/µL, with median values and range reported in parentheses.

^e^Distant disease was defined as sites beyond regional nodes.

### Response and toxicity

**Fig. 1:**
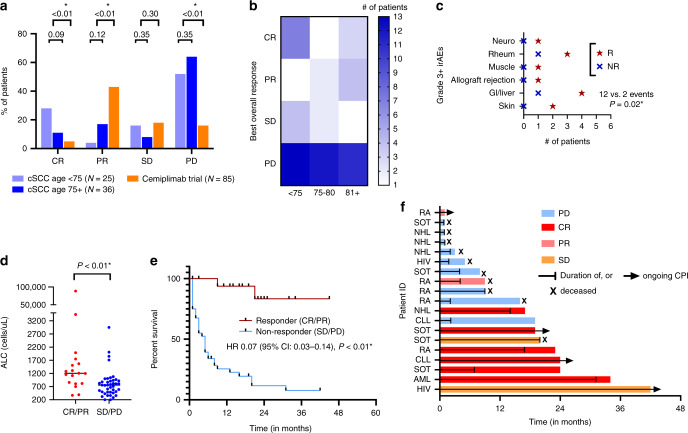
Real-world response to immune checkpoint inhibitors (CPI) among patients with advanced cutaneous SCC. **a** Bar graph comparing the best overall response (BOR) (as % of patients) observed in real-world cutaneous squamous cell carcinoma (cSCC) treated with immune checkpoint inhibitor (CPI) therapy compared to BOR in a landmark clinical trial (Migden et al.^6^), organised by age at initiation of CPI. Chi-square testing, *P < 0.05, two-sided. **b** Heatmap showing BOR rates (coded by colour intensity) among real-world cSCC patients grouped by age categories. **c** Grade 3+ immune-related toxicity (irAEs) by BOR to CPI. R   responders, NR   non-responders. Mann–Whitney test, *P < 0.05, two-sided. **d** Scatter plot showing pre-treatment absolute lymphocyte count (ALC) based on CPI response. Mann–Whitney test, *P < 0.05, two-sided. **e** Kaplan–Meier curve showing overall survival (in months) based on CPI response among real-world cSCC patients. Log-rank testing, *P < 0.05, two-sided. **f** Swimmer’s plot of time on CPI (in months) for *N* = 19 immunosuppressed, advanced cSCC patients. The *x* axis shows their respective immunosuppression/autoimmune disease. RA rheumatoid arthritis, SOT solid organ transplant, NHL non-Hodgkin lymphoma, HIV human immunodeficiency virus, CLL chronic lymphocytic leukaemia, AML acute myeloid leukaemia, PD progression, CR complete response, PR partial response, SD stable disease.

When comparing BOR to CPI therapy among cSCC patients in our cohort to previously published trial results,^6^ a significantly greater number of progressive disease (PD) events (59 versus 16.5%, *P* < 0.01) were observed with fewer objective responses (complete response [CR] + partial response [PR]) (31.5 versus 48%, *P* < 0.01) when pooling 1 L and 2 L exposure (Fig. 1a). Six patients (10%) were treated beyond first evidence of progression with none later achieving a confirmed response. Median duration of response (DOR) among our cohort was 20 months (range: 1–43+). BOR was similar for elderly cSCC patients compared with patients under 75 years of age (Fig. 1b), although the CR rate among patients <75 years of age was notable (28%). When assessing toxicity, rates of grade 3+ immune-related adverse events (irAEs) were overall similar to prior studies (20% versus 29–42%), and more common among responders when pooling all patients in the cohort (12 versus 2 events, *P* = 0.02), with gastrointestinal or hepatic toxicity most frequently observed (Fig. 1c). However, rates of grade 1–2 irAEs were similar, regardless of response (*P* = 0.63). Median ALC was higher among CPI responders (1205 versus 760 cells/uL, *P* < 0.01) (Fig. 1d), and response rates showed a non-significant trend towards favouring never versus former or current smokers (40 versus 22%, *P* = 0.13). In terms of the site of advanced disease, BOR rates were similar among locoregionally and distant disease subgroups (30.8 versus 36.3%, *P* = 0.18). In addition, BOR rates were similar when accounting for 1L versus 2L+ CPI use (29.8 versus 29.1%, *P* = 0.77).

For those who responded to a CPI, median OS (from the time of initiating CPI therapy) was significantly improved, as compared with non-responders (not reached [NR] versus 5 months, hazard ratio [HR] 0.07, 95% confidence interval [CI], 0.03–0.14, *P* < 0.01) (Fig. 1e).

Of the 19 immunosuppressed patients treated with CPI therapy, 8 (42%) experienced a response with a median DOR of 19.5 months (range: 1–32+), including 2/5 SOT recipients (both renal allografts) demonstrating a CR (with only 1/5 developing acute allograft rejection requiring haemodialysis) (Fig. 1f). Of the 19, 4 (21%) experienced grade 3+ irAEs while on CPI therapy. CPI response rates were somewhat lower among our immunocompetent patients (*n* = 42) comparatively, but this did not reach statistical significance (42 versus 31.5%, *P* = 0.32).

### Survival and comorbidity

**Fig. 2:**
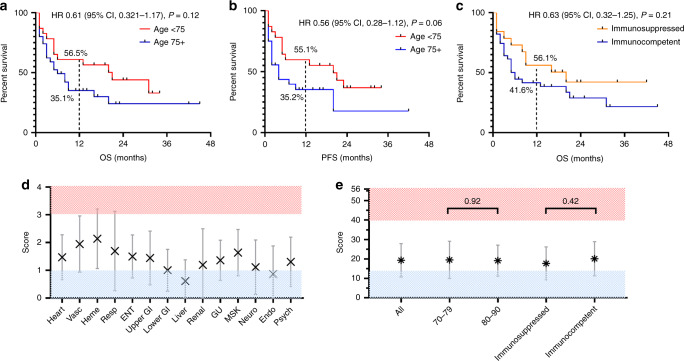
Survival among patients with advanced cutaneous SCC treated with an immune checkpoint inhibitor (CPI). Kaplan–Meier curves showing (**a**) overall survival (OS) in months and (**b**) progression-free survival (PFS) in months among advanced cSCC (cutaneous squamous cell carcinoma, cSCC) patients treated with an immune checkpoint inhibitor (CPI). **c** OS (in months) among advanced cSCC patients treated with a CPI based on immunosuppression history. **d** Cumulative Illness Rating Scale-Geriatric (CIRS-G) scores quantifying the burden of geriatric comorbidity among elderly (age 75 years or older) cSCC patients treated with a CPI, arranged by organ system. Mean values (X) and standard deviations (vertical bars) are displayed for each group. Scores in each category are 0–4 based on chronic disease burden (4 being the highest score in each category) at baseline in each organ system [Miller MD, et al.^12^; Kirkhus et al.^26^]. **e** Median CIRS-G composite score (maximum: 56) among elderly (age 75 years or older) cSCC patients treated with a CPI grouped by age and immunosuppression subgroups. Mann–Whitney comparison of ranks, *P < 0.05, two-sided.

With a median follow-up of 8.5 months (range: 0–45) among the entire cohort, the median OS was 8 months (95% CI, 7.6–12.3), with 46.1% of patients alive at 12 months. When comparing median OS by age cut-offs, findings were similar (*P* = 0.12) (Fig. 2a). Median PFS was 7 months (95% CI, 5.6–9.4), with 50.3% of patients alive and progression-free at 6 months. Median PFS was also similar, regardless of older age (*P* = 0.06), with 35.2% of patients 75 years and older alive and progression-free at 12 months (Fig. 2b). There was no OS difference based on immunosuppression history among cSCC patients from the start of CPI therapy (*P* = 0.21) (Fig. 2c).

In terms of organ system-specific (scale: 0–4) and cumulative (scale: 0–56) CIRS-G scores for the elderly subgroup, the lowest mean scores were in the liver category (0.61) and the highest mean in the haematologic (2.13) and vascular (1.94) comorbidity categories (Fig. 2d). The cumulative mean score for the entire cohort was 19.3 (range: 5–36), with no differences in cumulative mean scores by age subgroup (*P* = 0.92) or immunosuppression history (*P* = 0.42) (Fig. 2e). Beyond organ system function, Eastern Cooperative Oncology Group (ECOG) performance status was 0–1 among 80% of the elderly subgroup at the time of initiating CPI therapy.

When assessing the impact of individual clinicopathologic features on survival outcomes using multivariate analysis, a history of smoking (former or current) (HR 2.54, *P* = 0.01) was associated with poorer outcomes, while a higher pre-treatment ALC (HR 0.09, *P* = 0.02), response to a CPI (HR 0.06, *P* < 0.01) and the presence of a grade 3+ irAE (HR 0.19, *P* = 0.02) were all associated with improved survival (Supplementary Table 2).

### Tumour mutational burden

**Fig. 3:**
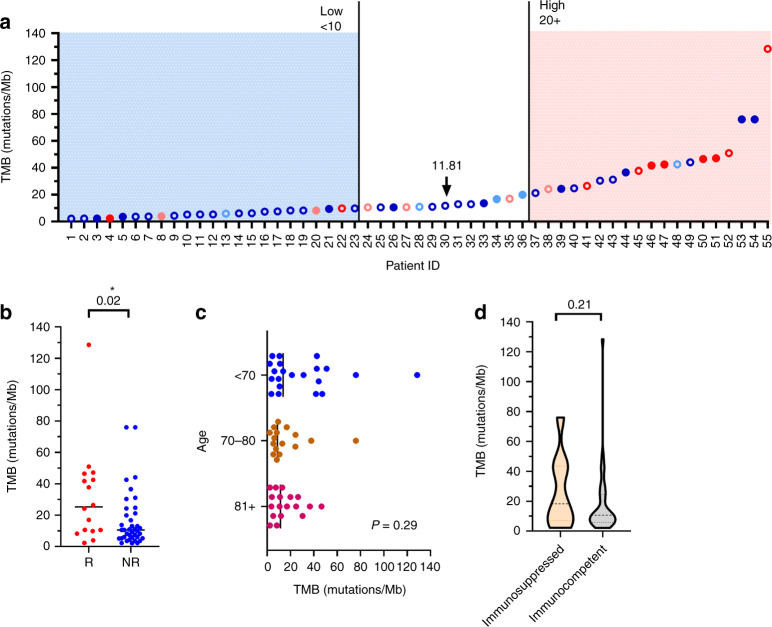
Tumour mutational burden (TMB) among advanced cutaneous SCC patients treated with an immune checkpoint inhibitor (CPI). **a** Tumour mutational burden (TMB) normalised as mutations per megabase (Mb) among advanced cutaneous SCC (cSCC) patients treated with an immune checkpoint inhibitor (CPI), arranged from the lowest to the highest TMB. Circle colours indicate the best overall response achieved (red = complete response, pink = partial response, light blue = stable disease and dark blue = progression) with immunotherapy for each patient. Filled-in circles identify immunosuppressed patients. The median TMB is indicated by an arrow and value on the graph. **b** Respective scatterplots showing median TMB values compared among responders (R) and non-responders (NR) to CPI, and **c** by patient age subgroups. **d** Violin plot comparing median TMB by immunosuppression history. Mann–Whitney comparison of ranks and Kruskal–Wallis test, ^*^P < 0.05, two-sided.

Fifty-five of 61 (90%) of our cSCC patients had tumour sequencing data available with a median TMB of 11.8 muts/Mb observed (Fig. 3a) with 19/55 (35%) having relatively high TMB values >20. Higher median TMB values were observed among cSCC patients who experienced a response to CPI therapy (25.4 versus 10.6 muts/Mb, *P* = 0.02) (Fig. 3b). Of note, TMB values were similar across all age subgroups in the cohort (*P* = 0.29) (Fig. 3c), and regardless of a patients’ history of immunosuppression (*P* = 0.21) (Fig. 3d).

## Discussion

### Lower overall response rates in real-world cSCC patients

The landmark trial using cemiplimab as PD-1 blockade in advanced cSCC was reported in 2018, showing a 48.2% overall response rate.^6^ This study included 64 (75.3%) patients ≥65 years of age (median age 71–73), but excluded those with significant immunosuppression or recent systemic therapy for autoimmune disease. When comparing these data to our real-world cohort, we observed lower BOR rates (31.5 versus 48%, *P* < 0.01) and higher rates of PD (59 versus 16.5%, *P* < 0.01) with more than half (66%) of our non-trial population over age 75 and/or immunosuppressed. Interestingly, age ≥75 and a history of immunosuppression did not individually account for the decrease in response observed. We recognise that we grouped patients with immune compromise or those with immunosuppression needs for the purposes of analysis, but that these patients exhibit a heterogeneous spectrum of immune competence that may be dynamic over time. While immunosenescence may play a role whereby elderly patients may have a lower capacity to mediate antitumour responses,^18^ several studies, including our own, show similar response rates to CPI among patients ≥65.^19,20^ This was despite our observed lower pre-treatment peripheral lymphocyte counts among our elderly subgroup.^21^ We speculate that there may be other biologic contributors to account for such findings, such as predominance of distant metastatic disease (77% of our cohort) or second primary diagnoses of cancer. Further, patient body weight,^22^ the concomitant use of medications such as antibiotics^23^ and corticosteroids^24^ may all negatively impact immunotherapy response and may have influenced our study findings.

Accounting for differences in BOR, median OS from the time of initiation of CPI therapy was lower than what has been reported in prior studies (estimated 12-month OS: 46.1 versus 81%), but with comparable estimated PFS rates at 12 months (43.6 versus 53%).^6^ Of interest, when comparing our cohort by age subgroups, there was no significant difference in median OS or PFS from the time of CPI initiation. Median OS was also similar among the immunosuppressed patients compared to the broader cohort (*P* = 0.21). We had hypothesised that the lower-than-predicted OS we observed was impacted by competing comorbidities, and so we explored cumulative mean CIRS-G comorbidity and ECOG performance scores. Scores were actually favourable among our elderly subgroup (75+ years of age), so we were not able to measure this difference if in fact, a difference in burden of comorbidity does exist. The CIRS-G is felt to be comprehensive and provides greater comorbidity detail and a higher sensitivity when compared to other morbidity indices.^25^ In multivariate analysis, former or current smoking predicted worse outcomes (HR 2.54, *P* = 0.01) among our cohort comprising 59% smokers. It is known that former smoking predicts more favourable outcomes to PD-1 blockade in non-small-cell lung cancer, which may relate to higher TMB and a tobacco gene signature,^26,27^ but this may not be the case for other tumour types where smoking may intensify comorbid health risks, and an ultraviolet (UV) gene signature may be more influential.

### Favourable response rates to CPI therapy among immunosuppressed patients

We observed favourable response rates (42%, 8/19) to CPI therapy across a broad range of medication- and disease-related immunosuppressive conditions, with a median DOR approaching 2 years (19.5 months, range: 1–32+). A patient with a history of haematopoietic stem cell transplant (>2 years out) for AML had a CR 2 months into CPI therapy with a 32-month disease-free interval that is ongoing, adding to a growing literature suggesting the safety of this approach.^28^ Two of five (40%) SOT recipients demonstrated a response (median DOR 20 months) with one exhibiting acute allograft rejection, despite high rates reported in small series.^29^ Of note, 3/5 SOT recipients were on mTOR inhibitors with prednisone (none were on antimetabolites or 3-drug regimens), which may have contributed to allograft preservation, and all were on doses of prednisone 20 mg daily or less. Despite these concerns, a 21% rate of grade 3+ irAEs was observed in the immunosuppressive subgroup, on par with immunocompetent cSCC patients, which may have resulted from overall modest immunosuppression use among our cohort. We should note that all our autoimmune patients had rheumatologic diseases that have been shown to have lower-grade immune exacerbations on CPI therapy.^30^

### irAEs, higher TMB and pre-treatment ALCs are predictors of response

A tendency for our cSCC patients to manifest off-target immune-mediated toxicity was associated with CPI response (*P* = 0.02). The correlation between irAEs and favourable response and/or survival has been increasingly reported in the literature in a wide variety of solid tumour types.^31–34^ This may reflect a propensity for the patient’s immune system to be malleable against both tumour- and self-antigens. Of note, those patients in our cohort with some of the longest duration of CPI benefit (22–32 months) had some of the most significant immune-mediated toxicity (renal allograft rejection and grade 4 encephalitis), but our cohort was not large enough to correlate specific organ system immunological involvement with outcomes. Early identification and steroid initiation likely explain the improved outcomes observed with these patients, as serious irAEs can prove fatal.

Broad sequencing efforts across tumour types have shown that high TMB predicts response to CPI,^35^ related to a high probability of tumour antigenicity and immune recognition; we found evidence of this in our population, as responders demonstrated a higher median TMB value (25.4 versus 10.6 muts/Mb, *P* = 0.02). This echoes the non-significant trend that was observed in the locally advanced cohort of the cemiplimab trial, although the absolute values were higher overall (74 versus 29 muts/Mb).^36^ Previously published sequencing data for immunotherapy-treated advanced cSCC patients of all ages from this institution (minimum age 52, *n* = 16; no overlap with the current cohort)^37^ showed a median TMB of 33 muts/Mb (range: 4–137), which was higher compared to our older cSCC cohort (33 versus 11.8 muts/Mb, *P* = 0.03). Other sequencing reports have included aggressive primary cSCC and lymph node metastases (not CPI treated) showing similarly higher median TMB values (among all coding regions) >60 muts/Mb^30,38,39^ in the absence of age restriction or mention of immunosuppression. We speculate that our results reflect a sampling bias, whereby 62% of sequenced cSCC cases were metastatic sites with biopsies obtained years after initial diagnosis and prior treatments; thus, TMB would be expected to be comparatively lower due to clonal selection and metastatic heterogeneity.

Similar to prior studies,^40^ we also observed that higher median ALC at the time of CPI initiation correlated with response (*P* < 0.01) with lower ALCs among our elderly subgroup (*P* = 0.03). Of note, there was a non-significant trend towards lower pre-treatment ALC among patients treated with CPI in 2L or greater compared with 1L (580 versus 800 cells/uL, *P* = 0.06) as would be expected with prior cytotoxic therapy exposure. ALC may serve as a surrogate for estimating the number of available lymphocytes circulating that could recapitulate the tumour microenvironment and possibly contribute to immunosurveillance.

## Conclusions

We demonstrate comparatively lower real-world response rates to CPI therapy with lower overall survival, even when accounting for older age and a history of immunosuppression. Despite these observations, CPI remains the standard of care for locoregionally advanced and incurable, metastatic cSCC, and overall response rates as well as tolerability are favourable with improved survival demonstrated among responders. Pre-treatment ALC, tumour mutational burden and the presence of immune-mediated toxicity appear to be biomarkers of CPI response. It is clear that immunotherapy agents have revolutionised the treatment of advanced skin cancers, but recognising distinct patterns of response among our oldest and most vulnerable patients has important implications for refining our therapeutic approach in the future.

## Supplementary information

Supplemental Tables

## Data Availability

Data used and/or analysed during this study are available from the corresponding author on reasonable request.
