# Supplementary material for: Real-world outcomes treating patients with advanced cutaneous squamous cell carcinoma with immune checkpoint inhibitors (CPI)
Source: Br J Cancer. 2020 Sep 1;123(10):1535–42. doi: 10.1038/s41416-020-01044-8 (PMC7653959; doi:10.1038/s41416-020-01044-8)
Supplement: Supplementary file 1 — Supplemental Tables [file 41416_2020_1044_MOESM1_ESM.docx]

**Supplemental Table 1.** **Patients with underlying immunosuppression or autoimmune conditions treated with an immune checkpoint inhibitor (CPI) for advanced cutaneous SCC**

| **ID** | **Gender** | **Condition** | **IS or ARVs while on CPI** | **Age at Dx^a^** | **Smoking** | **Histology** | **Initial Staging^b^** | **Initial Therapy for R/M Disease** | **Best Response to CPI^c^** | **DOR^d^**  **(months)** | **Grade 3+ irAEs^e^** |
| --- | --- | --- | --- | --- | --- | --- | --- | --- | --- | --- | --- |
| **298** | M | SOT | everolimus, prednisone | 48 | Y | SCC | IVA | CRT | PD |  |  |
| **230** | M | SOT | everolimus,  prednisone | 53 | N | SCC | IV | S + RT | CR | 18.0+ |  |
| **249** | M | CLL | -- | 58 | N | SCC | I | S + RT | CR | 22.0+ |  |
| **175** | M | HIV | ARVs | 60 | Y | SCC | IV | S + CRT | PD |  |  |
| **229** | M | CLL | -- | 65 | N | SCC | IVB | S + CRT | PD |  |  |
| **211** | M | SOT | sirolimus, prednisone | 66 | N | SCC | II | S | CR | 22.0+ | Y |
| **124** | M | AML | -- | 69 | N | SCC | IVA | CRT | CR | 32.0+ | Y |
| **59** | M | RA | -- | 75 | Y | SCC | IVA | CRT | PD |  |  |
| **75** | M | HIV | ARVs | 75 | N | SCC | IVA | IC + CRT | SD |  |  |
| **113** | M | NHL | -- | 75 | Y | SCC | IV | S + RT | PD |  |  |
| **135** | M | RA | -- | 75 | Y | SCC | II | S + RT | PD |  |  |
| **342** | M | RA | prednisone | 83 | Y | SCC | III | C | PR | 7.0 | Y |
| **293** | M | NHL | -- | 85 | Y | SCC | IV | CPI | CR | 15.0+ |  |
| **184** | M | SOT | tacrolimus, prednisone | 87 | Y | SCC | IVA | S + RT | SD |  | Y |
| **222** | F | RA | -- | 87 | Y | SCC | III | S | CR | 21.0+ |  |
| **399** | F | RA | methotrexate | 87 | Y | SCC | IVA | CPI | PR | 1.0+ |  |
| **359** | M | SOT | tacrolimus, prednisone | 89 | N | SCC | II | S + RT | PD |  |  |
| **28** | F | NHL | -- | 90 | N | SCC | III | S + RT | PD |  |  |
| **413** | M | NHL | -- | 95 | Y | SCC | IVA | RT | PD |  |  |

^a^ age at initial diagnosis of cutaneous squamous cell carcinoma (SCC), ^b^ American Joint Committee on Cancer (AJCC) Staging 7^th^ edition (2010), ^c^ as determined by RECIST v1.1, ^d^ duration of response (DOR) measured in months from the time of response confirmation to last follow-up (censored) without documentation of progression or death, ^e^ immune-related adverse events (irAEs) determined by CTCAE v5.0. ARVs = antiretrovirals, Dx = diagnosis, F = female, M = male, NHL = non-Hodgkin lymphoma, RA = rheumatoid arthritis, HIV = human immunodeficiency virus, SOT = solid organ transplant recipient (all kidney), CLL = chronic lymphocytic leukemia, AML = acute myeloid leukemia history with hematopoietic stem cell transplant, N = no prior tobacco use history, Y = yes to prior or current tobacco use, S = surgery, RT = radiotherapy, CRT = concurrent chemoradiotherapy, IC = induction chemotherapy, C = chemotherapy, CPI = immune checkpoint inhibitor, CR = complete response, PR = partial response, SD = stable disease, PD = progression of disease. ‘+’ indicates response is ongoing at last follow-up (censored).

**Supplemental Table 2.** **The impact of clinicopathologic features on survival among patients treated with an immune checkpoint inhibitor (CPI) for advanced cutaneous SCC**

| **Variable** | **Total (*N*=61)** | | |
| --- | --- | --- | --- |
|  | **HR** | **[95% CI]** | ***p*-value** |
| Age at initiation of CPI | 1.02 | 0.98-1.05 | 0.19 |
| Gender | 1.27 | 0.57-2.81 | 0.54 |
| Smoking history | 2.54 | 1.18-5.43 | **0.01** |
| Immunosuppression history | 0.63 | 0.30-1.32 | 0.22 |
| Primary tumor subsite^a^ | 1.46 | 0.98-2.05 | 0.71 |
| Initial stage of disease | 1.21 | 0.85-1.72 | 0.27 |
| Histologic differentiation^b^ | 1.19 | 0.76-1.87 | 0.33 |
| Site of advanced disease: locoregional or distant | 1.33 | 0.81-1.67 | 0.42 |
| Absolute lymphocyte count at baseline | 0.09 | 0.06-0.39 | **0.02** |
| CPI as first line systemic therapy | 0.97 | 0.48-1.94 | 0.93 |
| Response to CPI | 0.06 | 0.01-0.27 | **<0.01** |
| Presence of Grade 3+ Immune-related toxicity^c^ | 0.19 | 0.04-0.80 | **0.02** |
| Total mutational burden | 0.65 | 0.53-1.02 | 0.18 |

HR = hazard ratio, CI = confidence interval, CPI = immune checkpoint inhibitor. Multivariate Cox proportional hazard modeling was only performed if there were more than n=10 patients in each subgroup. ^a^ primary tumor subsite = head and neck vs. others (including unknown primary of cutaneous origin); ^b^ well or moderately vs. poorly differentiated; ^c^ as determined by CTCAE v5.0.
